# Testes-specific protease 50 promotes cell invasion and metastasis by increasing NF-kappaB-dependent matrix metalloproteinase-9 expression

**DOI:** 10.1038/cddis.2015.61

**Published:** 2015-03-26

**Authors:** Z B Song, J-S Ni, P Wu, Y L Bao, T Liu, M Li, C Fan, W J Zhang, L G Sun, Y X Huang, Y X Li

**Affiliations:** 1 National Engineering Laboratory for Druggable Gene and Protein Screening, Northeast Normal University, Changchun 130024, China; 2 Research Center of Agriculture and Medicine Gene Engineering of Ministry of Education, Northeast Normal University, Changchun 130024, China; 3 Department of Pathology, the First Hospital of Jilin University, Changchun 130041, China; 4 Institute of Genetics and Cytology, Northeast Normal University, Changchun 130024, China

## Abstract

The high mortality in breast cancer is often associated with metastatic progression in patients. Previously we have demonstrated that testes-specific protease 50 (TSP50), an oncogene overexpressed in breast cancer samples, could promote cell proliferation and tumorigenesis. However, whether TSP50 also has a key role in cell invasion and cancer metastasis, and the mechanism underlying the process are still unclear. Here we found that TSP50 overexpression greatly promoted cell migration, invasion, adhesion and formation of the stellate structures in 3D culture system *in vitro* as well as lung metastasis *in vivo*. Conversely, TSP50 knockdown caused the opposite changes. Mechanistic studies revealed that NF-*κ*B signaling pathway was required for TSP50-induced cell migration and metastasis, and further results indicated that TSP50 overexpression enhanced expression and secretion of MMP9, a target gene of NF-*κ*B signaling. In addition, knockdown of MMP9 resulted in inhibition of cell migration and invasion *in vitro* and lung metastasis *in vivo*. Most importantly, immunohistochemical staining of human breast cancer samples strongly showed that the coexpression of TSP50 and p65 as well as TSP50 and MMP9 were correlated with increased metastasis and poor survival. Furthermore, we found that some breast cancer diagnosis-associated features such as tumor size, tumor grade, estrogen receptors (ER) and progesterone receptors (PR) levels, were correlated well with TSP50/p65 and TSP50/MMP9 expression status. Taken together, this work identified the TSP50 activation of MMP9 as a novel signaling mechanism underlying human breast cancer invasion and metastasis.

Breast cancer is a leading cause of morbidity and mortality of women worldwide, and its invasion and metastasis is primarily responsible for breast cancer-diagnosed deaths. Tumor metastasis consists of a complex cascade of events, including cell adhesion, invasion and angiogenesis,^1^ and a group of proteolytic enzymes, which participate in the degradation of environmental barriers such as extracellular matrix and basement membrane are involved in this complicated processes.^2^ Among these enzymes, the matrix metalloproteinases (MMPs), which belong to a family of zinc-dependent endopeptidases, are collectively capable of degrading essentially all of the components of the extracellular matrix (ECM).^3, 4^

Among the MMPs, MMP9 (a 92 kDa type IV collagenase or gelatinase B) and MMP2 (a 72 kDa type IV collagenase or gelatinase A), which are highly expressed in various malignant tumors, have critical roles in the degradation of type IV collagen, and are considered to be associated with invasion and migration of tumor cell.^5, 6, 7, 8, 9^ MMP9 and MMP2 are upregulated in all human and animal tumors and appear to increase with developing stages of tumor progression.^10^ The basal levels of MMP9 in most cancer cell lines are usually low, and that its expression can be induced by treatment of PMA via the activation of transcription factors such as NF-*κ*B and AP-1,^11, 12^ whereas MMP2 is usually expressed constitutively.^13, 14^ Transcriptional regulation of MMP9 gene is frequently suggested to be mediated by an AP-1 regulatory element in their proximal promoter regions; however, other reports on the promoter of MMP9 suggest the involvement of NF-*κ*B transcription factor for the activation of MMP9.^15, 16^

*TSP50* is a testis-specific gene encoding a protein that is homologous to serine proteases. In addition to its expression in normal testes, *TSP50* is abnormally reactivated in many malignant breast tumor samples and colorectal carcinoma biopsies tested.^17, 18, 19^ Our previous studies revealed that knockdown of TSP50 inhibited cell proliferation and induced apoptosis in p19 and MDA-MB-231cell,^20, 21^ and overexpression of TSP50 efficiently promoted cell proliferation *in vitro* and stimulated tumor formation in nude mice. Mechanistic studies suggested that TSP50 could activate the NF-*κ*B signaling pathway by binding to the NF-*κ*B:I*κ*B*α* complex.^22^ Our recent data also showed that the threonine protease activity of TSP50 was required for its functions in hyperproliferation.^23^ All this observations suggested *that TSP50* participated in cell proliferation and tumorigenesis as an oncogene. However, whether *TSP50* also has a role in cell invasion and cancer metastasis, and the mechanism underlying the process are still unclear.

In this study, we report that TSP50 have the ability to promote cell invasion and cancer metastasis by increasing MMP9 expression through NF-*κ*B signaling pathway. Most importantly, we also show a strong correlation of TSP50 and MMP9 coexpression with the patient tumor metastasis. In summary, our results support a potentially important role for TSP50 in promoting human breast cancer metastasis.

## Results

### TSP50 promotes cell migration and invasion

**Figure 1:**
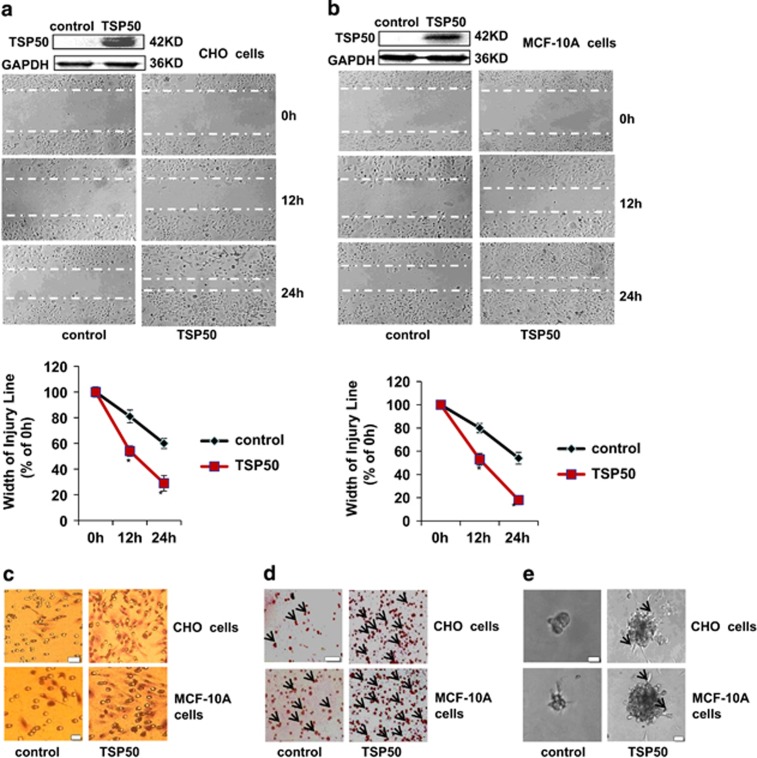
Effects of TSP50 overexpression on adhesion, invasion, migration and morphology of CHO and MCF-10A cells. (**a**) Top panel: expression of TSP50 in stably transfected CHO cells. Middle panel: overexpression of TSP50 promoted migration of CHO cells. Bottom panel: curve of different time intervals showing wound closure. The data for the migration assay represent three independent experiments. (**b**) Top panel: expression of TSP50 in stably transfected MCF-10A cells. Middle panel: overexpression of TSP50 promoted migration of MCF-10A cells. Bottom panel: curve of different time intervals showing wound closure. The experiments were performed as described in materials and methods. (**c**) Overexpression of TSP50 promoted invasion of CHO and MCF-10A cells. Control and TSP50-expressing CHO and MCF-10A cells were seeded in the upside of transwell coated with matrigel, after 40 h of incubation, the downward side of the membrane was stained with hematoxylin and eosin. Scale bar, 50 *μ*m. (**d**) Overexpression of TSP50 promoted adhesion of CHO and MCF-10A cells. The arrow points out the cells attached in the matrigel. Scale bar, 150 *μ*m. (**e**) Effects of TSP50 overexpression on the morphology of CHO and MCF-10A cells in three-dimensional (3D) cultures system as described in materials and methods. The arrow points out the filopodium. Scale bar, 50 *μ*m

To understand the biological function of TSP50 in cell mobility, we first explored the effects of TSP50 overexpression on cell migration and invasion *in vitro*. Results from wound healing assay suggested that TSP50-expressing CHO cells migrated much more rapidly compared with control cells, and almost fill the gap after 24 h (Figure 1a). Similarly, overexpression of TSP50 in MCF-10 cells also greatly promoted cell migration (Figure 1b). Likewise, we also observed that overexpression of TSP50 markedly enhanced the invasion of CHO and MCF-10A cells. As shown in Figure 1c, compared to TSP50-expressing CHO and MCF-10 cells, only a few control cells were capable of penetrating the Matrigel layer using a transwell chamber assay. The adhesion of cancer cells to ECM molecules is the first step of tumor cell invasion. Our results showed that overexpression of TSP50 significantly increased cells attached to matrigel (Figure 1d). We next investigated the effects of TSP50 overexpression on cell morphological character in 3D culture system. As shown in Figure 1e, compared with control, TSP50-expressing CHO and MCF-10A cells stretched out in the gel and formed many branched and elongated structures.

### Knockdown of endogenous TSP50 expression inhibits cell migration and invasion

**Figure 2:**
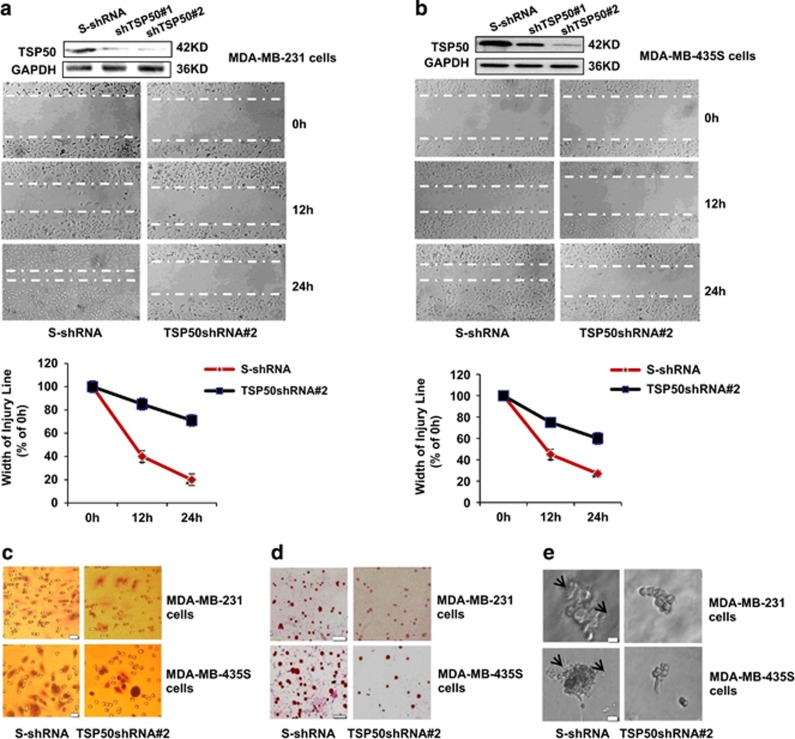
Silencing of endogenous TSP50 inhibited cell migration and invasion. (**a**) Top panel: western blotting assay of TSP50 expression in MDA-MB-231 cells transfected with scrambled shRNA and TSP50 shRNA vectors. Middle panel: knockdown of endogenous TSP50 inhibited migration of MDA-MB-231 cells. Bottom panel: curve of different time intervals showing wound closure. **(b)** Top panel: western blotting assay of TSP50 expression in MDA-MB-435S cells transfected with scrambled shRNA and TSP50 shRNA vectors. Middle panel: knockdown of endogenous TSP50 inhibited migration of MDA-MB-435S cells. Bottom panel: curve of different time intervals showing wound closure. (**c**) Knockdown of endogenous TSP50 inhibited invasion of MDA-MB-231 and MDA-MB-435S cells. Scale bar, 50 *μ*m. **(d)** Knockdown of endogenous TSP50 inhibited adhesion of MDA-MB-231 and MDA-MB-435S cells. Scale bar, 150 *μ*m. **(e)** Knockdown of endogenous TSP50 changed the morphology of MDA-MB-231 and MDA-MB-435S cells in three-dimensional (3D) cultures system. The arrow points out the filopodium. Scale bar, 50 *μ*m

To further analyze whether TSP50 was required for migration and invasion of breast cancer cells, we investigated the effect of the TSP50 downregulation on the migration and invasion of MDA-MB-231 and MDA-MB-435S cells. The MDA-MB-231 cells expressing TSP50 shRNA were established in our previous studies^22^ and were characterized again here (Figure 2a). We observed an effective suppression of wound healing in MDA-MB-231 cells with stable silencing of TSP50 expression (Figure 2a). Similarly, knockdown of TSP50 in MDA-MB-435S cells also greatly inhibited cell migration (Figure 2b). In addition, the invasion of MDA-MB-231 and MDA-MB-435S cells was also reduced by knockdown of endogenous TSP50 (Figure 2c). Likewise, cells attached to matrigel were also significantly reduced, and the branches disappeared and cells changed to spherical shape in 3D culture system when TSP50 expression was inhibited in both MDA-MB-231 and MDA-MB-435S cells (Figures 2d and e). Altogether, these data support the hypothesis of a critical role of TSP50 in promoting cell migration and invasion.

### The overexpression of TSP50 is crucial for the lung metastasis

**Figure 3:**
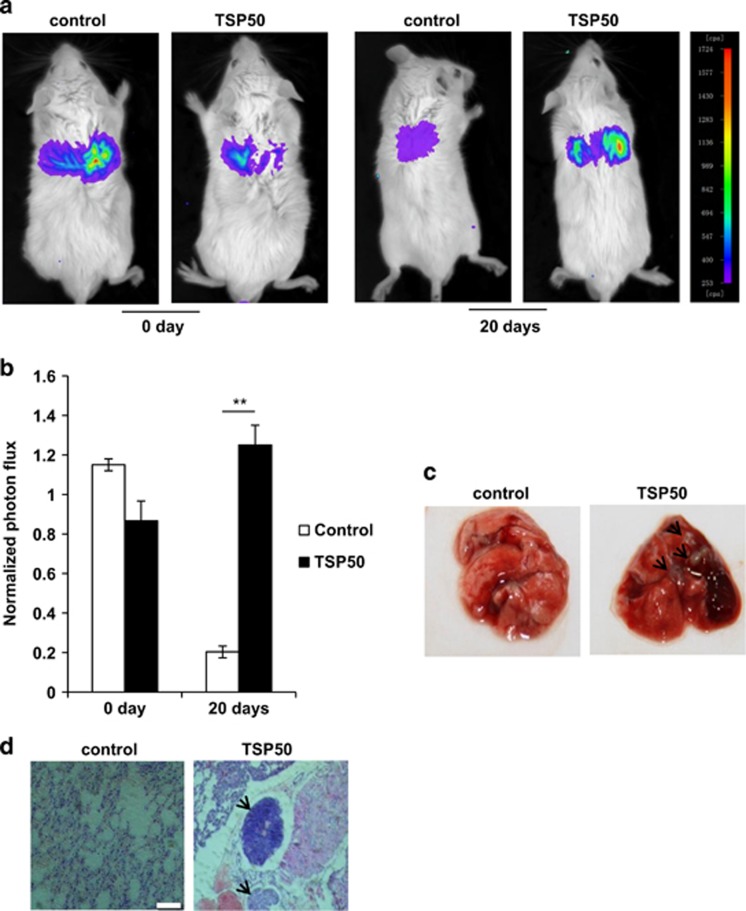
Overexpression of TSP50 promoted lung metastasis. (**a**) 2 × 10^6^ MCF-10A cells stably expressing control and TSP50 were injected through the tail vein of BALB/C mice. Mice were anaesthetized and injected with D-luciferin followed by bioluminescence imaging analysis of the lung metastasis as described in materials and methods section. (**b**) Quantification of bioluminescent imaging data. **P<0.01. (**c**) Appearance of the lungs from mice injected intravenously with control and TSP50-expressing cells. (**d**) Hematoxylin-eosin staining assay represent the metastases in the lungs. The arrow points out the metastasis nodules. Scale bar, 200 *μ*m

All the results described above pointed out a possibility that TSP50 expression was potentially important for the progression of tumor metastasis. To test this possibility, TSP50-expressing MCF-10A cells that had been engineered to stably express firefly luciferase were injected into BALB/C mice through tail veins and the lung metastasis of the cells was examined. Strikingly, the results showed that the presence of control cells in the lung was almost undetectable 20 days later, whereas cells stably expressing TSP50 succeed in metastasized to lung, suggesting that overexpression of TSP50 resulted in a noticeable increase in the metastasis (Figures 3a and b). To further confirm the tumor metastasis, we performed histological analyses of lung tissues from the mice. The hematoxylin and eosin (H&E) staining showed normal structure of lungs from control mice (without tumors), in contrast, lung tissues from mice injected with TSP50-expressing cells were heavily infiltrated by metastasized cells (Figures 3c and d). These results clearly demonstrate that TSP50 is critical in the regulation of the cell metastasis in a mouse model.

### NF-*κ*B signaling pathway is involved in TSP50-induced cell migration and invasion

**Figure 4:**
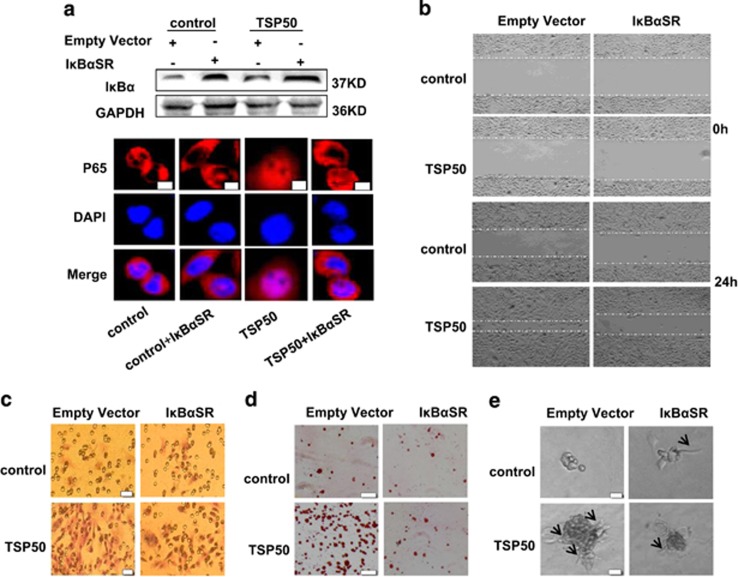
NF-*κ*B activation is required in TSP50-induced cell migration and invasion. (**a**) Top panel: Overexpression of I*κ*B-SR. Bottom panel: Overexpression of I*κ*B-SR suppressed TSP50-induced p65 nuclear translocation by immunofluorescence assay. (**b**) Overexpression of I*κ*B-SR suppressed TSP50-induced cell migration. Scale bar, 100 *μ*m. (**c**) Overexpression of I*κ*B-SR suppressed TSP50-induced cell invasion. Scale bar, 50 *μ*m. (**d**) Overexpression of I*κ*B-SR suppressed TSP50-induced cell adhesion. Scale bar, 150 *μ*m. (**e**) Effects of I *κ*B-SR expression on the morphology of TSP50-expressing cells. The arrow points out the filopodium. Scale bar, 50 *μ*m

Previously, we have demonstrated that TSP50 promoted cell proliferation and tumor formation through activation of NF-*κ*B signaling pathway.^22^ To determine whether NF-*κ*B activation was also necessary for TSP50-induced cell migration and invasion, a dominant-negative I*κ*B mutant (I*κ*B-SR) was transfected into control and TSP50-expressing CHO cells to block NF-*κ*B activation as described previously,^22^ and the expression of I*κ*B-SR and its effects on TSP50-induced p65 nuclear translocation was further determined (Figure 4a). As expect, cell migration of TSP50-expressing cells was reduced by I*κ*B-SR expression (Figure 4b). In addition, I*κ*B-SR expression greatly inhibited TSP50-induced cell invasion by transwell chamber assay (Figure 4c), suggesting that TSP50 required NF-*κ*B signal to enhance cell mobility. Likewise, TSP50-induced cell adhesion and the length of cell stellate structures in 3D medium were significantly reduced (Figures 4d and e). Taken together, these results suggest that NF-*κ*B activation is required in TSP50-induced cell migration and invasion.

### TSP50 enhanced MMP9 expression and activity

**Figure 5:**
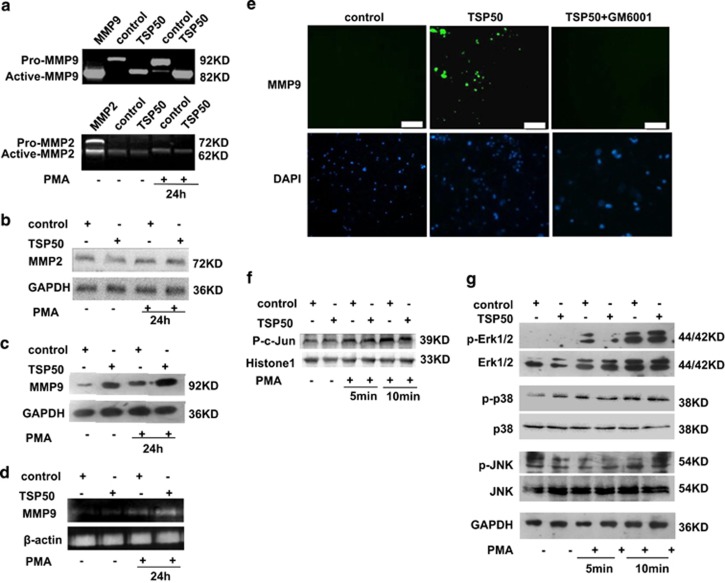
TSP50 promoted PMA-induced MMP9 expression. (**a**) The MMP2 and MMP9 activities in the media from either control or TSP50-expressing cells treated by PMA were examined by gelatin zymography as described in materials and methods. The activity of a recombinant MMP9 and MMP2 protein containing pro- and active band was used as a positive control. (**b**) Effects of TSP50 overexpression on MMP2 expression were analyzed by western bloting. (**c**) Effects of TSP50 overexpression on MMP9 expression were analyzed by western blotting. (**d**) Induction of MMP9 mRNA expression by TSP50. The MMP9 mRNA levels were measured by RT-PCR using *β*-actin as an internal control. (**e**) Increased *in situ* gelatinolytic activity by TSP50. The control and TSP50-expressing cells were processed for *in situ* zymography as described in materials and methods, and 20 mM of GM6001 was used to inhibit MMPS in TSP50-expressing CHO cells. (**f**) Effects of TSP50 expression on p-c-jun nuclear translocation. Scale bar, 150 *μ*m. (**g**) Effects of TSP50 expression on MAP kinase activities

Degradation of extracellular matrix and vascular basement membrane is required for invasion and metastasis of cancer, and MMP9 and MMP2 are critical proteinases that have such a role. Furthermore, MMP9 is a target of NF-*κ*B signaling pathway which was required in TSP50-induced cell migration and invasion as confirmed above. Therefore, we first tested the effect of TSP50 overexpression on PMA-induced MMP9 and MMP2 secretion by gelatin zymography assay. Our results showed that there was a dramatic increase of active MM9 secretion in the PMA-induced cells compared to control, and the PMA-induced MMP9 secretions were dramatically increased by TSP50 overexpression, while the level of MMP2 secretion was not affected by PMA or TSP50 overexpression (Figure 5a). Next, we further investigated the effect of TSP50 overexpression on MMP9 and MMP2 expression in protein level. Consistent with the results above, Western blotting assay suggested that the expression of MMP9 was significantly enhanced by overexpression of TSP50, while MMP2 expression was unaffected (Figures 5b and c). Furthermore, results from the RT-PCR also revealed that the expression of MMP9 was increased at transcription levels by TSP50 overexpression, in a similar manner with zymography analysis (Figure 5d). To confirm the role of TSP50 in activation of MMP9 for the cell invasion, we performed *in situ* zymography assay. We found that the gelatinolytic activity was increased in TSP50-overexpressing cells, however, when the MMPs inhibitor GM6001 was added, the increased gelatinolytic activity by TSP50 expression was inhibited (Figure 5e), indicating that TSP50 caused the matrix degradation by activation of MMP9.

It has been reported that AP-1 and the NF-*κ*B transcription factors are involved in the regulation of the *MMP9* gene expression.^12^ To further investigate whether AP-1 was also involved in the activation of the MMP9 transcription in TSP50-induced cell migration and invasion, we examined the effect of TSP50 overexpression on p-c-jun level in the nucleus and the mitogen-activated protein kinase (MAPK; ERK, JNK, p38) activities, which mediated the AP-1. As shown in Figure 5f, overexpression of TSP50 did not lead to a clear increase of p-c-jun nuclear translocation. Likewise, phosphorylation of the three types of MAPKs was not affected by TSP50 overexpression (Figure 5g), suggesting that TSP50 promoted MMP9 expression mainly through activation of NF-*κ*B signaling.

### Expression of MMP9 is crucial for the TSP50-induced cell invasion and lung metastasis

**Figure 6:**
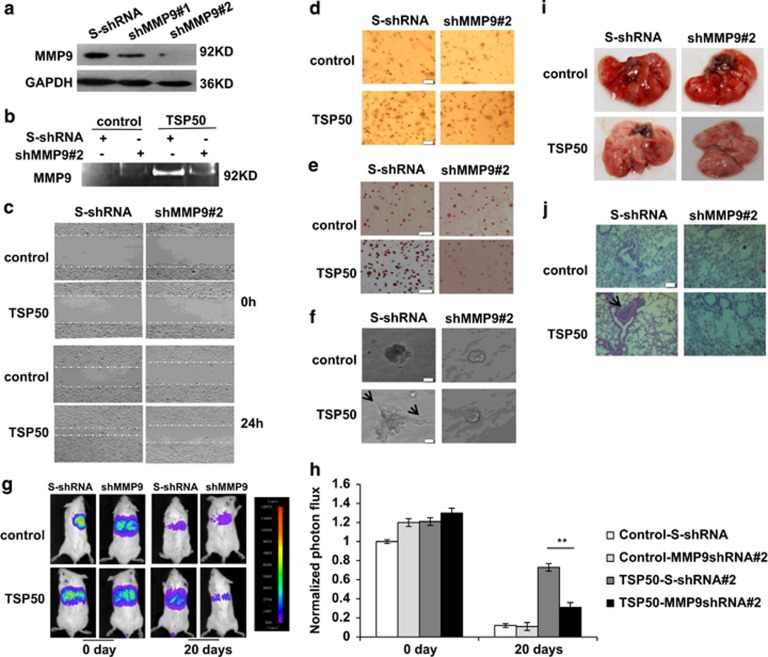
MMP9 is required for TSP50-mediated cell migration, invasion and metastasis. (**a**) western blotting assay of MMP9 expression in control and TSP50-expressing CHO cells transfected with scrambled shRNA and TSP50 shRNA vectors. (**b**) Knockdown of MMP9 decreased MMP9 activities by gelatin zymography. (**c**) Knockdown of MMP9 inhibited TSP50-induced cell migration. Scale bar, 100 *μ*m. (**d**) Knockdown of MMP9 inhibited TSP50-induced cell invasion. Scale bar, 50 *μ*m. (**e**) Knockdown of MMP9 inhibited TSP50-induced cell adhesion. Scale bar, 150 *μ*m. (**f**) Knockdown of MMP9 changed the morphology of TSP50-expressing CHO cells. The arrow points out the filopodium. Scale bar, 50 *μ*m. (**g**) The effect of MMP9 knockdown on lung metastasis of TSP50-expressing cells was examined by bioluminescence imaging analysis. (**h**) Quantification of bioluminescent imaging data. **P<0.01. (**i**) Appearance of the lungs from mice injected intravenously with control and MMP9-silencing cells. (**j**) Hematoxylin and eosin staining assay represent the metastases in the lungs. The arrow points out the metastasis nodules. Scale bar, 200 *μ*m

To further determine whether MMP9 was necessary for TSP50-induced cell migration and invasion, we analyzed the effect of MMP9 silencing on TSP50-induced cell migration and invasion. Two pRNAT-U6.1/Hygro expression vectors expressing shRNAs targeted against MMP9 were designed and named as shRNA#1, shRNA#2. Each was transfected into control and TSP50-overexpressing cells individually. The expression of MMP9 was analyzed by western blotting and gelatin zymography assay (Figures 6a and b). We found that knockdown of MMP9 resulted in a great decrease in the number of migrating cells into the wound area (Figure 6c), and similar results were obtained when cell invasion, adhesion and cell morphological character in 3D culture system were tested (Figures 6d–f).

For an in-depth understanding of the role of MMP9 in cell metastasis, we next extend our studies to an animal model. As shown in Figures 6g and h, the metastasis of TSP50-exressing cells to the lungs was significantly inhibited after knockdown of MMP9. Moreover, H&E analysis showed a difference in tumor nodule pattern distribution in lung histology sections (Figures 6g and h). Altogether, our results demonstrate that MMP9 is required for cell migration, invasion and metastasis mediated byTSP50.

### The positive correlation between TSP50 and MMP9 is correlated with patient tumor metastasis

**Figure 7:**
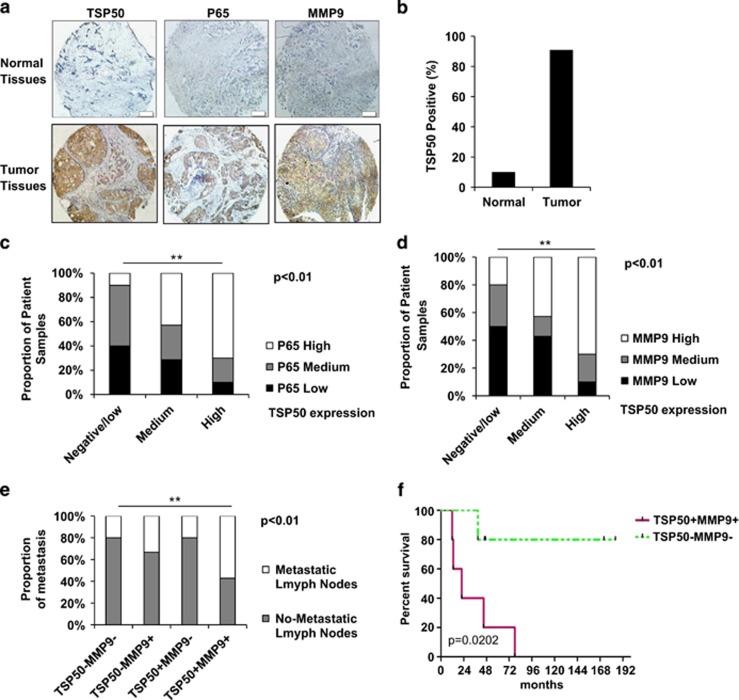
Co-overexpression of TSP50 and MMP9 is correlated with patient tumor metastasis. (**a**) Normal and tumor surgical specimens of human breast tumors (BR 1101) were subject to immunohistochemistry (IHC) using antibodies against TSP50, p65 and MMP9. Representative stains from the same tumor samples are shown. Scale bar, 100 *μ*m. (**b**) Analysis of TSP50 expression in human normal breast tissues (*n*=10) and human breast cancer tissues (*n*=88). (**c**) The correlation between TSP50 and p65 level was analyzed by Fisher's exact test. (**d**) The correlation between TSP50 and MMP9 level was analyzed by Fisher's exact test. (**e**) The correlation between the TSP50/MMP9 co-overexpression and the percentage of metastatic lymph nodes was analyzed by Fisher's exact test. (**f**) Kaplan–Meier plots of overall survival of breast cancer patients. Patient groups were separated based on expression status of TSP50 and MMP9 in a 30-sample data set (BR955). **P<0.01

The above results support a conclusion that TSP50 induces cell invasion and metastasis by promoting NF-*κ*B signaling and MMP9 expression. To further extend our findings *in vivo*, we determined whether there is a correlation of TSP50 with the activity of p65 and MMP9 in two tissue arrays of human breast cancer specimens. One of the arrays consists of 88 breast tumor specimens with 10 matched normal tissue counterparts, and the other is composed of 30 breast tumor specimens with survival data (Figure 7a). Among all specimens of 88 breast tumor specimens, 90.9% (80/88) of breast cancer tissues abundantly expressed TSP50 (Figure 7b), whereas only 1/10 of adjacent normal tissues stained positive for TSP50 protein. Moreover, accompanied by the increment of TSP50 protein levels in the 88 breast cancer tissues, nuclear level of p65 (Figure 7c) and expression of MMP9 (Figure 7d) markedly increased. Importantly, TSP50^+^/MMP9^+^ tumors were associated with a significantly higher metastasis rate, as shown in Figure 7e, 70/88 (79%) breast tumor specimens were positive for both TSP50 and MMP9, and 40/70(57%) of TSP50^+^/MMP9^+^ tumors showed one or more metastatic lymph nodes, whereas other tumors showed very fewer metastatic lymph nodes. Furthermore, among all specimens of the small group (30 breast tumor specimens) examined, TSP50^+^/MMP9^+^ tumors were also associated with a higher metastasis rate and poor patient survival (Figure 7f). Collectively, these results strongly suggest that the increase of MMP9 expression by TSP50 through NF-*κ*B signaling has a critical role in promoting human breast cancer invasion and metastasis.

### The positive correlation of TSP50 with p65 and MMP9 is a potential diagnostic marker for human breast carcinoma

**Figure 8:**
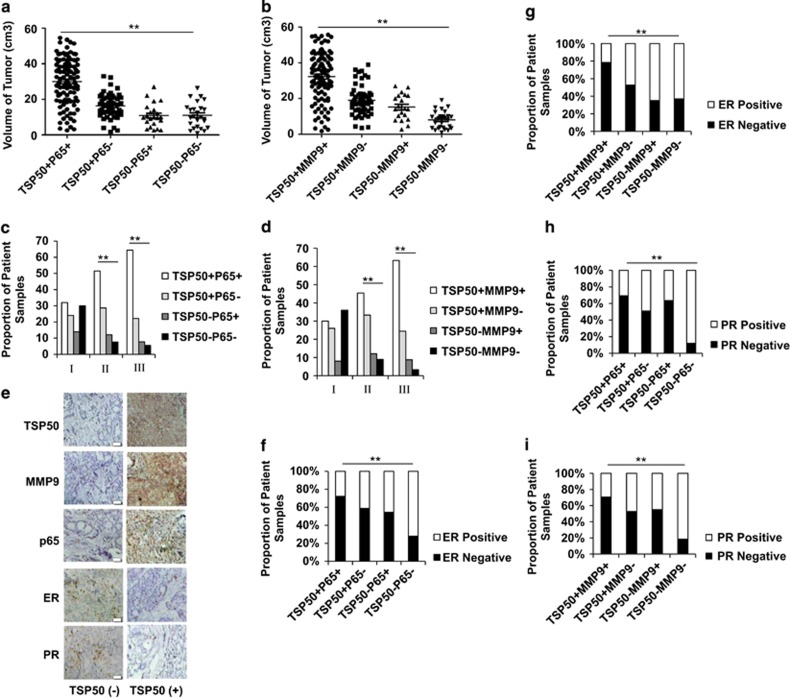
The correlation of TSP50/p65 and TSP50/MMP9 levels with some clinical and pathological parameters in human breast carcinoma. (**a**) The correlation between TSP50/p65 level and tumor sizes in the 206 breast cancer samples was analyzed by Fisher's exact test. Each data point represents an individual sample. (**b**) The correlation between TSP50/MMP9 level and tumor sizes in the 206 breast cancer samples was analyzed by Fisher's exact test. Each data point represents an individual sample. (**c**) The correlation between TSP50/p65 level and pathologic grade in the 206 breast cancer samples was analyzed by Fisher's exact test. (**d**) The correlation between TSP50/MMP9 level and pathologic grade in the 206 breast cancer samples was analyzed by Fisher's exact test. (**e**) Immunohistochemistry of TSP50, p65, MMP9, ER, and PR in the 206 breast cancer samples. (**f**) The correlation between TSP50/p65 level and ER expression was analyzed by Fisher's exact test. Scale bar, 200 *μ*m. (**g**) The correlation between TSP50/MMP9 level and ER expression was analyzed by Fisher's exact test. (**h**) The correlation between TSP50/p65 level and PR expression was analyzed by Fisher's exact test. (**i**) The correlation between TSP50/MMP9 level and PR expression was analyzed by Fisher's exact test. **P<0.01

On the basis of the above findings, we next investigated the clinical significance of TSP50/p65 and TSP50/MMP9 expression in another 206 human breast cancer samples. We first examined whether coexpression of TSP50 and 65 as well as TSP50 and MMP9 were correlated with tumor size and pathologic grade. Our data indicated that the TSP50^+^/p65^+^ or TSP50^+^/MMP9^+^ tumor were much larger than other tumors from the patients (Figures 8a and b). Similar results were obtained when the correlation of TSP50/p65 and TSP50/MMP9 expression with tumor grade was tested (Figures 8c and d). Especially, (58/90) 64% of breast tumors of pathologic grade III were TSP50^+^/p65^+^, and (57/90) 63% were TSP50^+^/MMP9^+^ (Supplementary Table1). As ERs and PRs could influence breast cancer prognosis, and testing the tumor for both estrogen and progesterone receptors is a standard part of a breast cancer diagnosis,^24^ we next analyzed whether expression of TSP50/p65 and TSP50/MMP9 were associated with these two clinical molecules in breast cancer patients (Figure 8e). As shown in Figures 8f and g, 72% (78/108) of TSP50^+^/ p65^+^tumors and 78% (80/102) of TSP50^+^/MMP9^+^ tumors were negative for ER expression. In contrast, 72% (18/25) of TSP50^−^/p65^−^tumors and 63% (17/27) of TSP50^−^/MMP9^−^ tumors were positive for ER expression (Supplementary Table 1), and the similar results were obtained when PR expression was examined (Figures 8h and i). Taken together, our results suggested that expression status of TSP50/p65 as well as TSP50/MMP9 may be taken as a potential diagnostic, prognosis and therapeutic marker for human breast carcinoma in early stage.

## Discussion

Breast cancer is the most prevalent cancer in the world, and the invasion and metastasis of tumor cells are the primary causes of morbidity and mortality.^25, 26^ *TSP50*, a novel oncogene overexpressed in breast cancer, has the potential to promote cell proliferation and tumorigenesis.^22^ In this study, we mainly focus on its function in cell invasion and metastasis.

In addtiton to the invasion-promoting function of TSP50, we also found it could change cell morphological character in 3D culture system (Figure 1e). *In vivo*, cells are surrounded by other cells and ECM, which formed a 3D environment. The traditional 2D monolayer culture system can not emulate the complexities of the 3D tissue microenvironment and always represent a suboptimal milieu for studying physiological ECM interactions with connective tissue cells. In contrast, the 3D cell culture models provide a well-defined *in vitro* microenvironment for studying the interactions of cell–ECM and cell–cell.^27, 28, 29^ Our data suggested that TSP50-overexpressing cells formed the filopodium in the 3D culture environment, and these structures migrated into the Matrigel to cleave the ECM with the help of the MMPs (Figures 1e and 2e).

Tumor invasion and metastasis are multistep and complex processes that include cell adhesion, proteolytic degradation of ECM, cell migration to basement membranes to reach the circulatory system, and remigration and growth of tumor at the metastatic site.^30^ Tumor cells adhesion to the ECM is a fundamental step in tumor invasion, the initial invasive action of metastatic cells involves the interaction of tumor with the ECM molecules through the process of cell matrix adhesion, which enhances the survival or invasiveness of tumor cells.^31, 32^ The results from our cell adhesion assay showed that TSP50 significantly increased cell adhesion to Matrigel compared with the control group, and knockdown of endogenous TSP50 expression markedly inhibited cell adhesion (Figures 1d and 2d). This result revealed that TSP50 promoted the invasion of breast cancer cells by increasing cell adherence to the basement membrane.

NF-*κ*B signaling pathway is constitutively activated in breast cancer, and has been shown to contribute to the development and progression of tumors.^33^ Several genes involved in angiogenesis, invasion and metastasis, such as VEGF, uPAR, MMP9, COX-2 and ICAM-1, have been identified as being regulated by NF-*κ*B.^34, 35, 36, 37, 38^ The frequent activation of NF-*κ*B in breast cancer cells suggests that breast tumor cells may acquire metastatic activity by overexpression of metastasis relevant genes during their progression. As we have previously demonstrated that TSP50 promoted cell proliferation by activating NF-*κ*B signaling pathway,^22^ we determined whether NF-*κ*B activation was also involved in TSP50-induced cell migration, invasion and metastasis. Our results indicated that inhibition of NF-*κ*B signaling greatly suppressed TSP50-induced cell migration, invasion and metastasis (Figure 4), suggesting that activation of NF-*κ*B signaling was required in this process.

MMPs form a family of zinc-dependent proteinases, and play a key role in the facilitation of cancer metastasis. MMPs have been implicated in primary and metastatic tumor growth and angiogenesis, and may even contribute to tumor promotion.^10, 39^ MMP9 and MMP2 play a key role in the degradation of type IV collagen that acts as the backbone of cellular basement membrane. While MMP2 is constitutively expressed in the tissues, MMP9, like the majority of other MMPs, can be stimulated to synthesize and secrete by a variety of stimuli including cytokines and PMA.^40^ Though many previous studies suggest that PMA induces MMP9 rather than MMP2 in various cell lines,^41, 42, 43^ the differential expression of MMP9 and MMP2 is likely to be related to the types of the stimuli and the cells.^40^ In the present study, we found that TSP50 overexpression significantly enhanced PMA-incuced MMP9 expression and secretion, however, it had no obvious effects on the level of MMP2 (Figures 5a and b). Given that MMP9 was a target of NF-*κ*B signal, we inferred that TSP50 promoted cell invasion and metastasis by enhancing MMP9 expression through NF-*κ*B signal, and our subsequent results demonstrated that MMP9 was required in TSP50-induced cell invasion and metastasis (Figure 6). However, we did not examine the role of other NF-*κ*B regulated genes, including ICAM-1 and VEGF, which are also involved in cell invasion and metastasis, and this may need further study.

Both TSP50 and MMP9 are aberrantly expressed in human breast cancer tissues, and here we found TSP50 overexpression promoted MMP9 expression through NF-*κ*B signaling pathway, therefore, we examined the correlation of TSP50 with p65 and MMP9 in human breast cancer clinical samples. We observed the positive relationship of TSP50 with p65 and MMP9 levels in breast cancer tissues (Figure 7). Moreover, we also found this positive relationship was associated with clinicopathological features, such as tumor sizes, pathologic grade, ER, and PR levels (Figure 8). These results strongly suggest that the upregulation of MMP9 through NF-*κ*B is an important signaling required for maximizing TSP50-promoted breast cancer metastasis.

Taken together, our results demonstrate that the TSP50 regulation of MMP9 expression through NF-*κ*B signaling is critical for human breast cancer cell invasion. Importantly, our results suggest a potentially significant correlation of TSP50 and MMP9 in the metastatic progression of human breast cancer, therefore, TSP50 may represent a novel favorable intervention target against breast cancer metastasis.

## Materials and Methods

### Antibodies and reagents

Polyclonal antibodies against MMP9, MMP2, I*κ*B*α*, P-c-Jun, JNK, p38 and p65 were obtained from Santa Cruz Biotechnology (Santa Cruz, CA, USA), and anti-GAPDH antibody was purchased from Kangcheng Biotech. Anti-Erk, p-Erk, p-p38 and p-JNK antibodies were purchased from Cell Signaling Technology (Danvers, MA, USA). An anti-TSP50 monoclonal antibody was prepared in our laboratory. PMA was purchased from Beyotime. MatrigelTM and MatrigelTM-coated filter inserts (8-*μ*m pore size) were obtained from Becton-Dickinson (Franklin Lakes, NJ, USA). MMPS inhibitor GM6001 was obtained from Chemicon (Chemicon International, Temecula, CA, USA). d-luciferin was from Beijing QWbio (Beijing, China).

### Cell lines and cell culture

CHO (Chinese hamster ovary cells), MDA-MB-231 (human breast cancer cells), MDA-MB-435S (human breast cancer cells) and MCF-10A (human mammary gland epithelial cells) were obtained from the Chinese Academy of Sciences Shanghai Institute for Biological Sciences-Cell Resource Center, which had characterized the cell lines by short tandem repeat profiling, cell morphology and karyotyping assay. CHO and MDA-MB-231 cells were cultured in Dulbecco's modified Eagle's medium (DMED, Gibco BRL, Rockville, MD, USA), MDA-MB-435S cells were cultured in Leibovitz's L-15 and MDA-MB-231 were cultured in DMEM/F-12 supplemented with 10% fetal bovine serum (TBD Science, Tianjin, China), 100 U/ml penicillin and 100 *μ*g/ml streptomycin (Invitrogen, San Diego, CA, USA), at 37 °C, 5% CO_2_. The TSP50-stable-expression CHO cell strain was obtained previously.^22^

### ShRNA preparation

The TSP50 and MMP9 shRNA expression vectors were prepared as described previously.^22^ The shRNA sequences targeting MMP9 were MMP9 shRNA#1 5′-CGTATCTGGAAATTCGACT-3′ and MMP9 shRNA#2 5′-CATCACCTATTGGATCCAA -3′.

### Western blotting assay

Western blotting was performed as described previously.^44^ Briefly, proteins resolved by SDS-PAGE were transferred to PVDF membranes, and blocked with 5% nonfat dry milk in TBST buffer (20 mM Tris-HCl pH 7.6, 150 mM NaCl and 0.05% Tween 20) for 1 h at room temperature. The membranes were then probed with diluted primary antibodies in 1% milk/TBST for 12 h at 4 °C, washed three times, incubated with HRP-conjugated secondary antibodies for 1 h at room temperature, and washed extensively before detection by chemiluminescence with ECL-Plus (Beyotime, Shanghai, China) and imaged by MicroChemi bio-imaging system (DNR, Jerusalem, Israel).

### Migration assay

Cells (5 × 10^5^) were seeded in six-well dishes and grown 90% confluence in 2 ml of growth medium. The cells were carefully scraped with a 5-mm-wide tip, and cellular debris was removed by washing with DMEM and cells were cultured in DMEM serum-free medium. Cell migration into the wound area was photographed at the indicated time points.

### Cell invasion assay

Cell invasion assay was performed as described previously.^45^ Briefly, cells to be tested for invasion were collected by trypsinization, washed and resuspended in conditioned medium, then added to the upper chamber of the Matrigel-coated invasion filter inserts (0.5 × 10^5^ cells/well). Conditioned medium without serum (600 *μ*l) was plated into the lower compartment of the invasion chamber. The chambers were incubated at 37 °C for 24 h in 5% CO_2_. After incubation for 40 h, the filter inserts were removed from the wells and the cells on the upper side of the filter were removed mechanically by wiping with a cotton swab. The filters were fixed for 10 min with paraform and stained with hematoxylin and eosin. The cells invading through the Matrigel were located on the underside of the filter. Photographs of three random fields were taken, and the number of cells was counted to calculate the average number of cells per field that had transmigrated.

### Gelatin zymography assay

Gelatin zymography assay was performed as described previously.^45^ Briefly, cells were incubated in serum-free DMEM with or without 200 nM PMA for a given time, and the conditioned medium was collected as samples. The samples unboiled were separated by electrophoresis on 8% SDS-PAGE containing 0.1% gelatin. After electrophoresis, the gels were washed twice in washing buffer (2.5% Triton X-100 in dH_2_O) at room temperature for 30 min to remove SDS, and then incubated in reaction buffer (10 mM CaCl_2_, 0.01% NaN_3_ and 40 mM Tris-HCl, pH 8.0) at 37 °C for 12 h to allow proteolysis of the gelatin substrate. Bands corresponding to the activity were visualized by negative staining using Coomassie Brilliant blue R-250 (Bio-Rad Laboratories, Richmond, CA, USA) and molecular weights were estimated by reference to prestained SDS-PAGE markers.

### *In situ* zymography

*In situ* gelatinolytic activity was detected using cell mmps zymography staining kit (GenMed Scientifics Inc, Shanghai, China) according to the manufacturer's instructions.

### Cell adhesion assay

The cell adhesion assay was performed as described previously.^46^ Briefly, the 96-well plates were coated with 5 *μ*g/ml Matrigel overnight, and nonspecific binding sites were blocked with 1% BSA in PBS for 4 h at 37 °C followed by washing three times with phosphate-buffered saline (PBS). Cells were trypsinized and resuspensed in DMEM serum-free medium, and 3 × 10^4^ cells/well were added to each coated well. The cells were incubated at 37 °C for 1 h and the nonadherent cells were removed by shaking the plate at 500 g for 30 s and washing with PBS three times, and then cells were fixed for 10 min with paraform and stained with hematoxylin and eosin.

### Three-dimensional (3D) culture

The 3D culture model was established as described previously.^47^ Briefly, 1 × 10^4^ cells were suspended with 100 *μ*l 10 *μ*g/ml Matrigel and kept at 37 °C until gelled. The plates were then incubated at 37 °C and cultures were grown for 7–10 days before being observed under microscope.

### RNA Extract and RT-PCR

The RNA Extract and RT-PCR were performed as described previously.^23^ Total RNA (3 *μ*g) was reverse transcribed into cDNA by incubating at 50 °C for 60 min. The cDNA was amplified by PCR with the following primers: MMP9: 5′-CACTGTCCACC CCTCAGAGC-3′ (sense) and 5′-GCCACTTGTCGGCGATAAGG-3′ (antisense); *β*-actin: 5′-CAAGAGATGGCCACGGCTGCT-3′ (sense) and 5′-TCCTTCTGCATCCTGTCGGCA-3′ (antisense). PCR was performed for 30 cycles (each cycle consisting of 94 °C for 30 s, 54 °C for 30 s and 72 °C for 30 s). The PCR products were analyzed by electrophoresis on a 1% agarose gel stained with GeneGreen and visualized under UV light.

### Immunofluorescence detection

Cells were seeded on sterile coverslips in six-well tissue culture plates. After 24 h, cells were fixed in 3.7% formalin and washed three times with PBS. Nonspecific sites were then blocked with PBS containing 5% bovine serum albumin (BSA) for 30 min at room temperature. Thereafter, anti-p65 antibody was flooded over the cells, and the cultures were incubated at 4 °C overnight. After washing with PBS, the cells were further incubated with Cy3-conjugated goat anti-rabbit IgM for 1 h at room temperature, followed by washing with PBS and then analyzed using an Olympus BX50 fluorescence microscope (Olympus, Tokyo, Japan).

### Bioluminescence imaging analysis of lung metastasis

Female Balb/c nude mice 6–8-weeks old were used for all xenografting studies. For lung metastasis formation, 2 × 10^6^ cells were washed and collected in 0.2 ml PBS and subsequently injected into the lateral tail vein. Mice were anaesthetized using isoflurance and injected intraperitoneally with 150 mg/kg of d-luciferin. Imaging was completed with *in vivo* Imaging System (NightOWL LB 983, Berthold, Germany). Mice are imaged at day 0 and day20. At the end of experiment, animals are then processed for subsequent histology. All animal work was done in accordance with a protocol approved by the Institutional Animal Care and Use Committee and all experiments conform to the relevant regulatory standards.

### Immunohistochemistry

The clinical breast cancer tissue microarrays (BR955 and BR 1101 from US Biomax, Rockville, MD, USA US Biomax) were analyzed as described previously.^23, 48^ Another 206 cases of human breast cancer tissue samples were collected from the First Hospital of Jilin University with the informed consent of patients. Tumor tissues were fixed and paraffin embedded. Sections (5 *μ*m) were cut, dewaxed, rehydrated and subjected to Immunohistochemistry. The study was approved by the ethics committee of Northeast Normal University, and written informed consent was obtained from all patients whose samples were collected.

### Statistical analysis

All values were repeated at least three times. Data were expressed as mean±SD. Statistical analysis of the data was performed using the Student's *t*-test and two-tailed distribution. The Fisher's exact test was performed to determine the association of TSP50 with p65 or MMP9 levels as well as the clinicopathological feature in human breast cancer samples.

## Abbreviations

AP: activator protein
CHO cell: Chinese hamster ovary cell
ERK: extracellular signal-regulated kinase
ECM: extracellular matrix
ER: estrogen receptors
GAPDH: glyceraldehyde 3-phosphate dehydrogenase
H&E: hematoxylin and eosin
JNK: c-Jun N-terminal kinase
MMP: matrix metalloproteinase
MAPK: mitogen-activated protein kinase
NF-*κ*B: nuclear factor *κ*B
PR: progesterone receptors
PMA: Phorbol myristate acetate
RT-PCR: reverse transcription-PCR
TSP50: testes-specific protease 50
