# Supplementary material for: Testes-specific protease 50 promotes cell invasion and metastasis by increasing NF-kappaB-dependent matrix metalloproteinase-9 expression
Source: Cell Death Dis. 2015 Mar 26;6(3):e1703–. doi: 10.1038/cddis.2015.61 (PMC4385939; doi:10.1038/cddis.2015.61)
Supplement: Supplementary Table S1 [file cddis201561x1.doc]

**Table S1**

**a**

**Positive correlation between co-expression of TSP50 and P65 with some clinical and pathological parameters in human breast carcinoma**

**Average tumor volume**

**(cm^3^)**

**Clinical Stages ER level PR level**

Ⅰ Ⅱ Ⅲ - + - +

**TSP50^+^/P65^+^ 16 34 58 78 30 75 33 32.9**

**TSP50^+^/ P65 –  12 19 20 30 21 26 25 16.4**

**TSP50^-^/ P65 ^+^  7 8 7 12 10 14 8 10.9**

**TSP50^-^/ P65 -  15 5 5 7 18 3 22 11.0**

b

**Positive correlation between co-expression of TSP50 and MMP9 with some clinical and pathological parameters in human breast carcinoma**

**Average tumor volume**

**(cm^3^)**

**Clinical Stages ER level PR level**

Ⅰ Ⅱ Ⅲ - + - +

**TSP50^+^/MMP9^+^ 15 30 57 80 22 72 30 32.3**

**TSP50^+^/MMP9 –  13 22 22 30 27 30 27 18.7**

**TSP50^-^/MMP9^+^  4 8 8 7 13 11 9 14.9**

**TSP50^-^/MMP9^-^  18 6 3 10 17 5 22 7.8**
